# Remnant cholesterol is independently asssociated with an increased risk of peripheral artery disease in type 2 diabetic patients

**DOI:** 10.3389/fendo.2023.1111152

**Published:** 2023-02-15

**Authors:** Yi Song, Ying Zhao, Xiangli Bai, Wenzhuo Cheng, Li Wang, Meng Shu, Yan Shu, Liyin Zhang, Si Jin

**Affiliations:** ^1^ Department of Endocrinology, Institute of Geriatric Medicine, Liyuan Hospital, Tongji Medical College, Huazhong University of Science and Technology, Wuhan, China; ^2^ Geriatric Medicine Center, Key Laboratory of Endocrine Gland Diseases of Zhejiang Province, Department of Endocrinology, Zhejiang Provincial People’s Hospital, Affiliated People’s Hospital, Hangzhou Medical College, Hangzhou, China; ^3^ Department of Laboratory Medicine, Liyuan Hospital, Tongji Medical College, Huazhong University of Science and Technology, Wuhan, China

**Keywords:** type 2 diabetes, peripheral artery disease, remnant cholesterol, risk factor, lipid

## Abstract

**Background:**

Remnant cholesterol (RC) has been correlated with a higher risk of atherosclerosis. It has been confirmed that in the general population, an elevated RC level is related to a 5-fold higher risk of peripheral arterial disease (PAD). Diabetes is one of the strongest risk factors for PAD development. However, the association between RC and PAD in the specific population of type 2 diabetes mellitus (T2DM) has not been investigated. Herein, the correlation was investigated between RC and PAD in T2DM patients.

**Methods:**

In the retrospective study, the hematological parameter data of 246 T2DM patients without PAD (T2DM - WPAD) and 270 T2DM patients with PAD (T2DM - PAD) was collected. Differences in RC levels between the two groups were compared, and the association between RC and PAD severity was examined. Multifactorial regression was used to determine whether RC was a significant contributor to the development of T2DM - PAD. The diagnostic potential of RC was tested using receiver operating characteristic (ROC) curve.

**Results:**

The RC levels in T2DM - PAD individuals were considerably greater than in T2DM - WPAD individuals (*P* < 0.001). RC had a positive correlation with disease severity. Further, multifactorial logistic regression analyses found that elevated RC levels were a major contributor to T2DM - PAD (*P* < 0.001). The area under the curve (AUC) of the RC for T2DM - PAD patients was 0.727. The cut-off value of RC was 0.64 mmol/L.

**Conclusion:**

The RC levels were higher in T2DM - PAD patients, and were independently linked with its severity. Diabetic patients with RC levels > 0.64 mmol/L had an elevated risk of developing PAD.

## Introduction

PAD is a chronic arterial occlusive disease of the lower limbs caused by atherosclerosis and is linked with substantial disability and death (1). T2DM is a main factor in the progression of atherosclerosis. The incidence of PAD rises in tandem with the occurrence of T2DM (2). In addition, diabetic people have a worse prognosis for PAD than non-diabetic ones (1). Thus, prompt diagnosis and treatment of PAD in diabetic subjects are necessary to reduce the danger of major adverse limb events (MALEs) (2).The ankle-brachial index (ABI) is currently recommended as the primary screening tool for PAD in diabetic patients and those with multiple risk factors (3). The ABI’s limited sensitivity in detecting PAD in its earliest stage highlights the critical need to discover new markers that may detect PAD in diabetics at an earlier stage.

RC is the cholesterol in triglyceride-rich lipoproteins and consists of very low-density lipoproteins (VLDL), intermediate-density lipoproteins (IDL), and chylomicron remnants (4). RC-level assessment can be easily calculated using established formulas, which are easy-to-access, and may provide valuable data for clinical management (5). Evidence from large prospective cohort studies based on the general population suggests a causal relationship between high remnant cholesterol levels and cardiovascular disease(CVD), and it is well established that lowering these lipoproteins reduces atherosclerotic cardiovascular events in humans (6–8). Recent studies have confirmed the atherogenic potential of RC, however, many of these studies focused on elevated RC levels in coronary arterial disease (CAD) and cerebrovascular disease, demonstrating an association between elevated RC levels and the risk of ischemic heart disease, myocardial infarction, and ischemic stroke (8–10). Interestingly, a recent investigation showed that in the general population, an elevated RC level was associated with a five-fold higher risk of PAD, greater than for myocardial infarction and ischemic stroke (10). High RC levels are common in diabetic individuals and has been linked to atherosclerosis through lipid metabolism and insulin resistance (11). It’s intriguing to speculate about whether or not RC also plays a part in the development of PAD in diabetics. Nevertheless, until now, there has been no study on whether there is a correlation between RC and PAD in T2DM population. The aim of the research was to examine whether higher RC levels were related to higher PAD risk among T2DM individuals.

## Materials and methods

### Study population

The cross-sectional research involved 514 gender-matched diabetic patients consecutively admitted to the Department of Endocrinology and Metabolism of the Liyuan Hospital affiliated to Tongji Medical College, Huazhong University of Science and Technology (Wuhan, China), from 1 March 2018 to 30 October 2022. T2DM patients with or without PAD were recruited. T2DM was defined as a fasting plasma glucose(FPG) level ≥ 7.0 mmol/L and/or 2-h plasma glucose(PG) ≥11.1 mmol/L during OGTT and/or HbA1c level ≥ 6.5%, based on the T2DM international criteria (ADA) (12). The inclusion criteria were patients aged 18-79 years with a confirmed diagnosis of T2DM. The excluding criteria were: a) coronary artery disease (CAD); b) history of stoke; c) diabetic retinopathy; d) acute complications of diabetes mellitus (such as diabetic ketoacidosis, hyperglycemia hyperosmotic state, and lactic acidosis); e) chronic kidney disease with an estimated glomerular filtration rate (eGFR) less than 60 mL/min; f) documented liver cirrhosis with Child–Pugh C dysfunction; g) history of active solid or hematological malignancy or autoimmune diseases; h) ABI > 1.4; i)RC < 0; j) suspected or confirmed pregnancy; k) undefined type of diabetes or clinical suspicion of non-type 2 diabetes mellitus; l) previous non-traumatic lower limb amputation; m) incomplete clinical data.

Each patient included in the study was evaluated for a history of PAD symptoms. The ABI was measured in patients with PAD-like symptoms. ABI was calculated according to the Transatlantic Inter-Society Consensus Document II (TASC-II) guidelines for the management of peripheral arterial disease (13). ABI was calculated as the ratio of ankle-to-brachial artery systolic pressure. ABI was computed by dividing the highest systolic pressure recorded in either the right or left brachial arteries or the anterior or posterior tibial arteries in each limb (14). The physician evaluated the patients’ lower extremities using arterial Doppler-enhanced ultrasonography if they had symptoms in both legs. Patients with an ABI > 0.90 who were asymptomatic were not additionally evaluated for PAD.

Patients whose ABI < 0.9 underwent arterial Doppler-enhanced ultrasonography of the limb extremities. The common femoral artery, femoral artery bifurcation, popliteal artery, posterior tibial artery, and dorsalis pedis artery were examined. The evaluation and score of vascular pathology were as follows: a) Artery intima thickness: normal (< 1 mm), 0 point; moderately thickened (1 – 1.2 mm), 1 point; severely thickened (> 1.2 mm), 2 points. b) Hardening: normal, 0 point; mildly hardened (the intima was not thickened, the echo was increased, and with no plaque), 1 point; moderately to severely hardened (mildly hardened, accompanied with plaque or stenosis), 2 points. c) Plaque: normal (no plaque forming), 0 point; single plaque, 1 point; numerous plaques, 2 points; scattered plaques, 3 points. d) Stenosis: normal, 0 point; mild stenosis (narrowing by 30%–50%), 1 point; moderate or severe stenosis (narrowing by 50% – 75%), 2 points; occlusion (no blood flow), 3 points. The degree of PAD was categorized based on the total number of points: a) 0 point, normal; b) < 10 mild; c) 10 – 20 points, moderate; d) > 20 points, severe (15).

### Demographic and clinical assessment

Demographic variables (age and gender), as well as laboratory results, such as blood count, total cholesterol (TC), high-density lipoprotein cholesterol (HDL-C), were obtained from the electronic medical record system in Liyuan hospital. On the second hospital morning, blood samples were collected from all patients’ peripheries. Laboratory personnel unaware of the patient’s diagnoses analyzed the blood samples.

RC levels were determined as TC (mmol/L) minus LDL-C (mmol/L) and HDL-C (mmol/L), as recommended by the dyslipidemia guidelines (16). The triglyceride glucose index (TyG index), neutrophil to lymphocyte ratio (NLR), monocyte to lymphocyte ratio (MLR), and platelet to HDL-C ratio (PHR) were calculated using the following formulas: TyG index = Ln [Triglyceride (TG, mg/dl) × FPG (mg/dl)/2]; NLR = neutrophil (10^9^/L)/lymphocyte (10^9^/L); MLR = monocyte (10^9^/L)/lymphocyte (10^9^/L); PHR = platelet (10^9^/L)/HDL-C (mmol/L).

### Statistical analysis

Statistical analyses were done using SPSS version 27.0 software (SPSS, Inc., Chicago, IL, United States). Graphs were created using Prism 9.0 (GraphPad Software). The normality of continuous variables was examined by the Shapiro-Wilk test. Continuous variables were described as means ± SDs and evaluated utilizing the Student’s t-test (two groups) or the One-way ANOVA (three groups). Non-normally distributed continuous variables were described as medians (interquartile ranges) and assessed using the Mann-Whitney U test (two groups) or Kruskal-Wallis test (three groups). Categorical variables were described as the numbers and percentages of patients. Chi-square tests were performed to assess categorical variables. The link between RC and PAD phases was analyzed by utilizing spearman correlation and partial correlation analysis. The relationship between RC and other variables in PAD patients was analyzed by Spearman correlation analysis. Covariates were excluded from the correlation analysis. Univariate and multivariate logistic regression analysis were used to examine the association between RC and PAD. The optimal value for identifying the risk of PAD in this sample was calculated using ROC curve analysis. The optimal cutoff value was determined by maximizing the Yoden index. Statistical significance was defined as a two-sided *P* value < 0.05.

## Results

### Comparison of baseline clinical features and laboratory indicators between the PAD group and WPAD group

The demographic and clinical data of T2DM - PAD group and T2DM - WPAD group are summarized in **Table 1**. Among the 516 diabetic patients enrolled, 270 had PAD, and 246 did not (WPAD). Compared to WPAD patients, PAD patients had a higher prevalence of hypertension (*P* < 0.05), and showed significantly increased levels of age, diabetes duration, systolic blood pressure (SBP), urea, creatinine (Cr), C-reactive protein (CRP), RC, neutrophils, monocytes, NLR, MLR, and PHR (*P* < 0.05), and showed significantly decreased levels of diastolic blood pressure (DBP), alanine aminotransferase (ALT), eGFR, HDL-C, and lymphocytes (*P* < 0.05). The two groups did not differ for gender, history of smoking, drinking, and dyslipidemia, aspartate aminotransferase (AST), uric acid, FPG, glycosylated hemoglobin (HbA1c), TG, TC, LDL-C, non-HDL-C(N-HDL-C), TyG index, and platelets (*P* > 0.05). Significant differences in glucose-lowering measures and statin use were found between the two groups (both *P* < 0.05). The incidence of mild, moderate, and severe PAD was 50.7, 23.3, and 25.9% in PAD patients, respectively.

**Table 1:** Demographic and clinical data of diabetic subjects with and without PAD.

Variables	WPAD	PAD	*P* value
	(N=246)	(N=270)	
Gender (male, %)	135 (54.9%)	156 (57.8%)	0.507
Age (years)	**57 (50-62)**	**65 (59-71)**	<**0.001**
Diabetes duration (years)	**5 (1-10)**	**10 (5-18)**	<**0.001**
Smoking, n (%)	69 (28%)	73 (27%)	0.797
Alcohol, n (%)	60 (24.4%)	68 (25.2%)	0.839
Hypertension, n (%)	**120 (48.8%)**	**167 (61.9%)**	**0.003**
Dyslipidemia, n (%)	90 (36.6%)	92 (34.1%)	0.551
SBP (mmHg)	**127 (117-138)**	**132 (123-144)**	<**0.001**
DBP (mmHg)	**80 (72-86)**	**77 (70-85)**	**0.028**
ALT, U/L	**20.4 (14.7-30.7)**	**17 (12.1-23.1)**	<**0.001**
AST, U/L	20 (16-25.6)	18.5 (15.8-24.5)	0.140
Urea, mmol/L	**5.53 (4.49-6.38)**	**5.9 (4.53-7.2)**	**0.006**
Cr, UMOL/L	**62.4 (51.5-77.4)**	**70.7 (57.3-85.6)**	<**0.001**
eGFR (ml/min/1.73m^2^)	**101.7 (91.3-110.9)**	**91.1 (72.2-103.7)**	<**0.001**
Uric Acid, μmol/L	309.8 (252.3-363.3)	319.4 (246.1-378.2)	0.461
CRP, mg/L	**1.2 (0.7-2.4)**	**2.1 (1.1-5.5)**	<**0.001**
FPG, mmol/L	9.98 (7.42-14.8)	9.99 (7.86-14.6)	0.796
HbA1c (%)	8.1 (6.7-9.9)	8.2 (7.2-9.8)	0.320
TG, mmol/L	1.55 (1.08-2.21)	1.66 (1.14-2.4)	0.275
TC, mmol/L	4.54 (3.92-5.32)	4.47 (3.64-5.41)	0.701
HDL-C, mmol/L	**1.14 (0.96-1.38)**	**1 (0.86-1.15)**	<**0.001**
LDL-C, mmol/L	2.82 (2.06-3.44)	2.52 (1.91-3.27)	0.053
N-HDL-C, mmol/L	3.38 (2.69-4.13)	3.36 (2.62-4.22)	0.899
RC, mmol/L	**0.55 (0.38-0.7)**	**0.75 (0.6-1.03)**	<**0.001**
TyG index	7.84 (7.25-8.48)	7.92 (7.35-8.49)	0.498
Neutrophil,10^9^/L	**3.43 (2.83-4.46)**	**3.98 (3.11-5.11)**	<**0.001**
Lymphocyte, 10^9^/L	**1.69 (1.41-2.03)**	**1.47 (1.13-1.85)**	<**0.001**
Monocyte, 10^9^/L	**0.34 (0.27-0.41)**	**0.38 (0.3-0.49)**	<**0.001**
Platelet, 10^9^/L	207 (178-243)	206 (171-258)	0.841
NLR	**2.05 (1.59-2.61)**	**2.66 (1.89-3.74)**	<**0.001**
MLR	**0.19 (0.16-0.25)**	**0.26 (0.19-0.35)**	<**0.001**
PHR	**179.26 (143.06-237.76)**	**204.08 (163.08-272.73)**	<**0.001**
Use antidiabetes agents			
Insulin, n (%)	**27 (11%) ^a^**	**60 (22.2%) ^b^**	**<0.001**
Oral drugs, n (%)	**143 (58.1%) ^a^**	**117 (43.3%) ^b^**	
Diet control only, n (%)	**41 (16.7%) ^a^**	**21 (7.8%) ^b^**	
Insulin + Drugs, n (%)	**35 (14.2%) ^a^**	**72 (26.7%) ^b^**	
Statins use, n (%)	**29 (11.8%)**	**55 (20.4%)**	**0.008**
PAD			
Mild PAD, n (%)	/	137 (50.70%)	
Moderate PAD, n (%)	/	63 (23.30%)	
Severe PAD, n (%)	/	70 (25.90%)	

SBP, systolic blood pressure; DBP, diastolic blood pressure; ALT, alanine aminotransferase; AST, aspartate aminotransferase; Cr, creatinine; eGFR, estimated glomerular filtration rate; CRP, C-reactive protein; FPG, fasting plasma glucose; HbA1c, glycosylated hemoglobin; TG, triglyceride; TC, total cholesterol; HDL-C, high-density lipoprotein cholesterol; LDL-C, low-density lipoprotein cholesterol; N-HDL-C, non-HDL-C; RC, **remnant cholesterol;** TyG index, triglyceride glucose index; NLR, neutrophil to lymphocyte ratio; MLR, monocyte to lymphocyte ratio; PHR, platelet/HDL-C ratio. P <0.05 (two-sided) was defined as statistically significant. Bold values indicate statistically significance. a, b: after applying the chi-square test, different superscripts indicate statistically different categorical variables between the 2 groups.

### Clinical and laboratory features of T2DM - PAD patients: Subgroup analysis according to PAD severity

The three groups did not differ regarding gender, duration of diabetes, history of smoking, drinking, and dyslipidemia, SBP, DBP, and laboratory parameters such as ALT, AST, urea, Cr, FPG, HbA1c, TC, LDL-C, N-HDL-C, TyG index, neutrophils, lymphocytes, monocytes, NLR, and MLR (*P* > 0.05) (**Table 2**). As disease severity increased, history of hypertension, eGFR, and HDL-C presented a decreasing trend (*P* < 0.05), but TG, RC, platelets, and PHR showed an increasing trend (*P* < 0.05). Moderate PAD patients had the highest levels of age, uric acid, and CRP (*P* < 0.05). Significant differences were found between the three groups using only oral medication or only insulin (*P* < 0.05).

**Table 2:** Demographic and clinical data of T2DM – PAD group according to PAD severity.

Variables	Mild PAD (N=137)	Moderate PAD (N=63)	Severe PAD (N=70)	*P* value
Gender (male, %)	82 (59.9%)	37 (58.7%)	37 (52.9%)	0.619
Age (years)	**64 (59-69)**	**68 (60-71)**	**67 (59-72)**	**0.043**
Diabetes duration (years)	10 (5-17)	10.5 (5-18)	10 (5-20)	0.447
Smoking, n (%)	39 (28.5%)	19 (30.2%)	15 (21.4%)	0.456
Alcohol, n (%)	43 (31.4%)	12 (19%)	13 (18.6%)	0.058
Hypertension, n (%)	**73 (53.3%)**	**43 (68.3%)**	**51 (72.9%)**	**0.011**
Dyslipidemia, n (%)	54 (39.4%)	15 (23.8%)	23 (32.9%)	0.093
SBP (mmHg)	130 (122-142)	132 (120-140)	135 (124-148)	0.093
DBP (mmHg)	77 (70-85)	76.5 (69-81)	78 (74-86)	0.230
ALT, U/L	17.9 (13.7-24.1)	16.2 (11.3-23.1)	15 (10.7-21.9)	0.115
AST, U/L	18.7 (16.3-24.1)	18.4 (15.6-25.5)	18.2 (15-25.5)	0.881
Urea, mmol/L	5.85 (4.51-6.8)	6.04 (4.89-7.41)	5.99 (4.45-7.89)	0.472
Cr, UMOL/L	70.1 (57.2-81.2)	71.25 (60.5-92.7)	71.3 (55.9-88.6)	0.253
eGFR (ml/min/1.73m^2^)	**93.9 ± 19.71**	**90.1 ± 20.97**	**89.15 ± 20.32**	**0.042**
Uric Acid, μmol/L	**304.3 (233.9-353.1)**	**337.15 (259.1-391.5)**	**335.25 (261.7-395.8)**	**0.021**
CRP, mg/L	**1.7 (0.9-4.1)**	**2.65 (1.3-10.4)**	**2.55 (1.4-10.6)**	**<0.001**
Fasting glucose, mmol/L	10.52 (8.21-15.51)	9.95 (7.62-14.06)	8.74 (6.98-13.37)	0.060
HbA1c (%)	8.7 (7.2-10.2)	7.8 (6.9-9.6)	7.95 (7.2-9.1)	0.209
TG, mmol/L	**1.53 (1.09-2.09)**	**1.78 (1.14-2.71)**	**1.89 (1.22-2.9)**	**0.046**
TC, mmol/L	4.47 (3.63-5.44)	4.39 (3.49-5.19)	4.67 (3.82-5.25)	0.613
HDL-C, mmol/L	**1.01 (0.9-1.25)**	**0.97 (0.83-1.12)**	**0.94 (0.77-1.11)**	**0.005**
LDL-C, mmol/L	2.67 (1.97-3.55)	2.49 (1.86-3.23)	2.35 (1.84-3.03)	0.186
N-HDL-C, mmol/L	3.26 (2.51-4.27)	3.42 (2.62-4.06)	3.5 (2.72-4.23)	0.824
RC, mmol/L	**0.68 (0.54-0.87)**	**0.8 (0.67-1)**	**1.05 (0.75-1.45)**	**<0.001**
TyG index	7.90 ± 0.84	7.98 ± 0.84	7.99 ± 0.92	0.692
Neutrophil,10^9^/L	3.73 (3.06-5.02)	3.96 (3.1-4.7)	4.305 (3.37-5.36)	0.142
Lymphocyte, 10^9^/L	1.51 (1.2-1.87)	1.37 (1.06-1.78)	1.44 (1.14-1.85)	0.154
Monocyte, 10^9^/L	0.38 (0.3-0.48)	0.37 (0.26-0.52)	0.41 (0.33-0.54)	0.258
Platelet, 10^9^/L	**200 (167-247)**	**207 (171-248)**	**227.5 (183-312)**	**0.032**
NLR	2.4 (1.81-3.56)	2.85 (2.06-3.93)	2.82 (2.19-3.79)	0.075
MLR	0.25 (0.19-0.34)	0.27 (0.19-0.38)	0.28 (0.21-0.37)	0.138
PHR	**185.53 (151.54-252.33)**	**211.42 (163.08-308.57)**	**247.45 (182.65-315.46)**	**<0.001**
Use antidiabetes agents				
Oral drugs	**68 (49.6%) ^a^**	**27 (42.9%) ^a,b^**	**20 (28.6%) ^b^**	**0.013**
Insulin	**18 (13.1%) ^a^**	**17 (27%) ^a,b^**	**23 (32.9%) ^b^**	
Insulin + drugs	37 (27%) ^a^	15 (23.8%) ^a^	22 (31.4%) ^a^	
Diet control only	14 (10.2%) ^a^	4 (6.3%) ^a^	5 (7.1%) ^a^	
Statins use	29 (21.2%)	11 (17.5%)	15 (21.4%)	0.806

SBP, systolic blood pressure; DBP, diastolic blood pressure; ALT, alanine aminotransferase; AST, aspartate aminotransferase; Cr, creatinine; eGFR, estimated glomerular filtration rate; CRP, C-reactive protein; FPG, fasting plasma glucose; HbA1c, glycosylated hemoglobin; TG, triglyceride; TC, total cholesterol; HDL-C, high-density lipoprotein cholesterol; LDL-C, low-density lipoprotein cholesterol; N-HDL-C, non-HDL-C; RC, **remnant cholesterol;** TyG index, triglyceride glucose index; NLR, neutrophil to lymphocyte ratio; MLR, monocyte to lymphocyte ratio; PHR, platelet/HDL-C ratio. P < 0.05 (two-sided) was defined as statistically significant. Bold values indicate statistically significance. a, b: after applying the chi-square test, different superscripts indicate statistically different categorical variables between the 3 groups.

**Figure 1:**
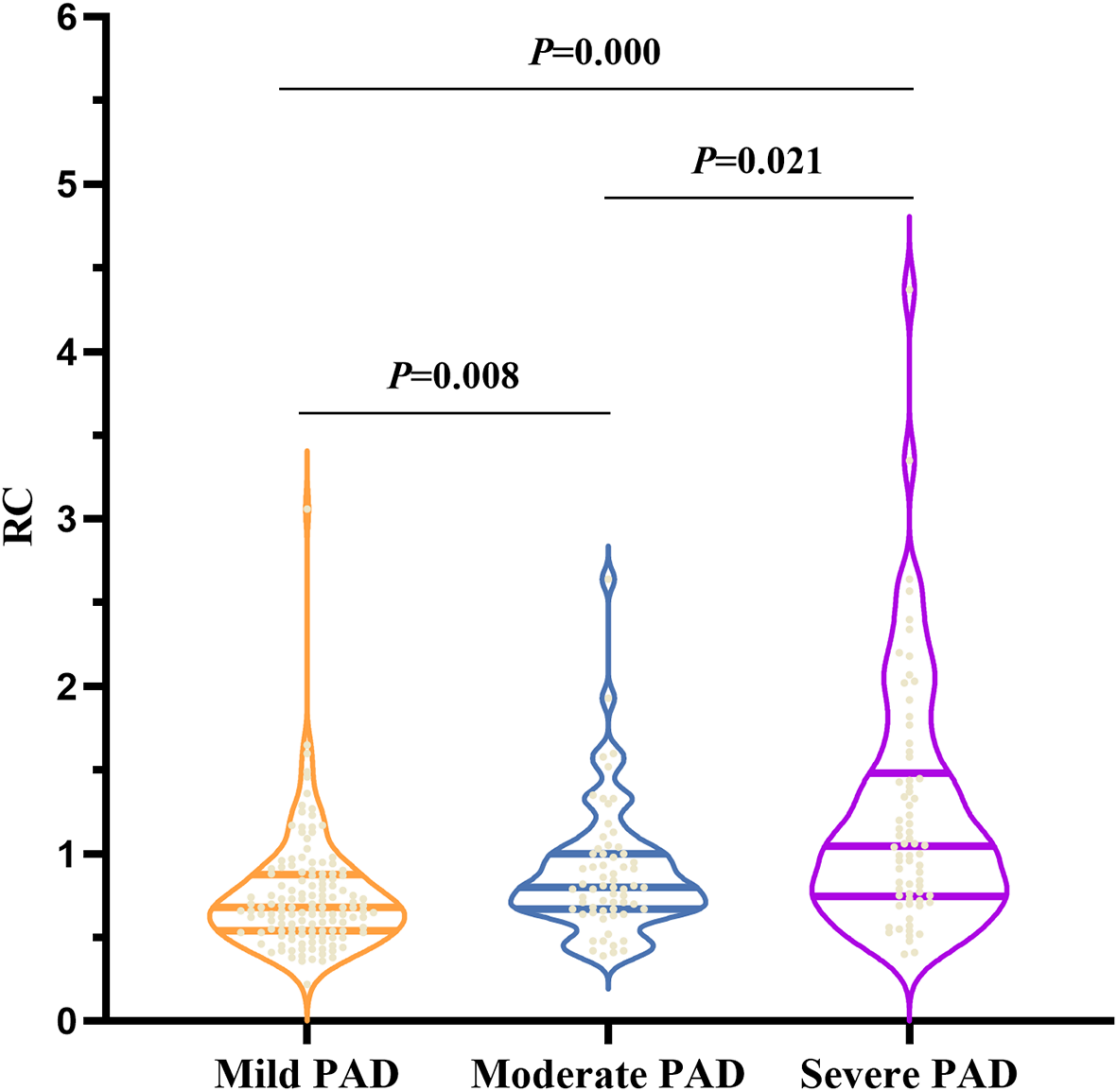
The RC levels according to PAD severity based on ultrasound results. RC, **remnant cholesterol.** In the violin plot, the three horizontal lines from top to bottom represent the upper quartile, the median, and the lower quartile in order. *P* < 0.05 (two-sided) was defined as statistically significant. Bold values indicate statistically significance.

The violin - plot in **Figure 1** found that the RC levels showed an increasing relationship with disease extent.

### Correlation of RC and other lipid variables with severity levels of T2DM – PAD

The correlations between RC and other lipid variables were assessed by utilizing spearman correlation analysis (including TG, TC, LDL-C, HDL-C, and N-HDL-C) in PAD patients. Based on the data in **Table 3**, RC (r = 0.387, *P*  <  0.001), TG (r = 0.151, *P* = 0.013), and HDL-C (r = -0.197, *P* < 0.001) were associated with the PAD severity levels. RC still maintained connections with PAD stages after adjusting for TG and/or HDL-C using partial correlation analysis.

**Table 3:** The correlation between stages of T2DM – PAD and the following lipid profiles.

Variables	Spearman Correlation Analysis		Partial Correlation Analysis	
	r	*P* value	r	*P* value
RC, mmol/L	**0.387**	**<0.001**	–	–
TG, mmol/L	**0.151**	**0.013**	**0.371^a^**	**<0.001**
TC, mmol/L	0.020	0.738	**0.416^b^**	**<0.001**
LDL-C, mmol/L	-0.111	0.069	**0.389^c^**	**<0.001**
HDL-C, mmol/L	**-0.197**	**<0.001**	**0.384^d^**	**<0.001**
N-HDL-C, mmol/L -	0.036 -	0.555 -	**0.410^e^** **0.388^f^**	**<0.001** **<0.001**

RC, remnant cholesterol; TG, triglyceride; TC, total cholesterol; HDL-C, high-density lipoprotein cholesterol; LDL-C, low-density lipoprotein cholesterol; N-HDL-C, non-HDL-C. Associations between serum lipid profile and stages of PAD by Spearman correlation analysis and the association between RC and stages of DR by partial correlation analysis a: Adjusted for TG; b: Adjusted for TC; c: Adjusted for LDL-C; d: Adjusted for HDL-C; e: Adjusted for N-HDL; f: Adjusted for TG and HDL-C. P < 0.05 (two-sided) was defined as statistically significant. Bold values indicate statistically significance.

### Univariate and multivariate logistic regression analysis of RC for T2DM - PAD occurrence

As univariate logistic regression analysis showed (**Table 4**), age, duration of diabetes, history of hypertension, SBP, DBP, ALT, urea, Cr, eGFR, HDL-C, CRP, RC, NLR, MLR, and PHR were independently associated with PAD occurrence in T2DM patients (*P* < 0.05). After excluding the effects of confounding factors for multivariate logistic regression, age, duration of diabetes, HDL-C, RC, NLR, MLR, and PHR were still statistically significant. RC, NLR, MLR, and PHR were considered independent risk factors for PAD occurrence in T2DM patients, while HDL-C was an independent protective factor.

**Table 4:** Univariate and binary logistic regression analysis results.

	Variable OR (95% CI)	*P* value	Variable OR (95% CI)	*P* value
Age	1.123 (1.096-1.150)	<0.001	**1.125 (1.086-1.167)**	**<0.001**
Diabetes duration	1.128 (1.095-1.162)	<0.001	**1.104 (1.063-1.147)**	**<0.001**
Hypertension	1.702 (1.199-2.417)	0.003		
SBP	1.019 (1.009-1.03)	<0.001		
DBP	0.981 (0.965-0.997)	0.019		
ALT	0.975 (0.962-0.988)	<0.001		
Urea	1.144 (1.045-1.252)	0.004		
Cr	1.01 (1.002-1.018)	0.011		
eGFR	0.97 (0.96-0.979)	<0.001		
HDL-C	0.178 (0.096-0.333)	<0.001	**0.141 (0.059-0.337)**	**<0.001**
CRP	1.059 (1.029-1.089)	<0.001		
RC	9.41 (5.1-17.363)	<0.001	**12.653 (6.112-26.197)**	**<0.001**
NLR	1.647 (1.394-1.945)	<0.001	**1.288 (1.032-1.608)**	**0.025**
MLR	1.795 (1.483-2.173)	<0.001	**1.568 (1.211-2.03)**	**<0.001**
PHR	1.004 (1.002-1.006)	<0.001	**1.006 (1.003-1.009)**	**<0.001**

SBP, systolic blood pressure; DBP, diastolic blood pressure; ALT, alanine aminotransferase; Cr, creatinine; eGFR, estimated glomerular filtration rate; CRP, C-reactive protein; HDL-C, high-density lipoprotein cholesterol; RC, remnant cholesterol; NLR, neutrophil to lymphocyte ratio; MLR, monocyte to lymphocyte ratio; PHR, platelet/HDL-C ratio. P < 0.05 (two-sided) was defined as statistically significant. Bold values indicate statistically significance.

### Diagnostic performance of RC for T2DM - PAD

**Figure 2:**
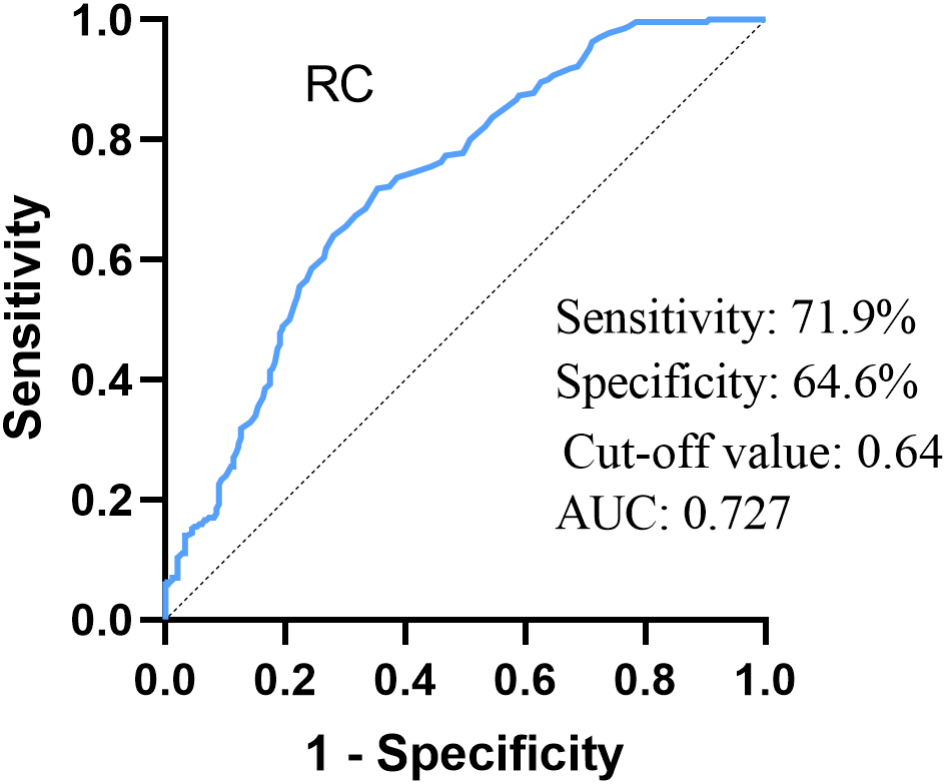
ROC curve analysis of the ability of RC to predict T2DM – PAD. RC, remnant cholesterol. AUC = 0.727, 95% CI:0.683–0.770, P = 0.000, cut-off: 0.64, sensitivity 71.9%, specificity 64.6%.

The ability of RC to identify T2DM - PAD patients was evaluated by the ROC curve. **Figure 2** showed that RC exhibited a high predicting value for T2DM – PAD (AUC = 0.727). The optimum RC cut-off value for predicting the occurrence of PAD in the group was 0.64 mmol/L (Sensitivity 71.9%, Specificity 64.6%).

### Correlation of RC with other parameters of T2DM - PAD patients

Correlations between RC and other indicators in PAD patients were assessed using Spearman correlation analysis. The RC had a significant and positive correlation with gender (r = 0.330), fasting glucose (r = 0.125), TG (r = 0.641), TC (r = 0.342), N-HDL-C (r = 0.379), TyG index (r = 0.485), and PHR (r = 0.123) (all *P* < 0.05) (**Table 5**).

**Table 5:** Correlation of RC with other potential risk factors in the T2DM-PAD patients.

Variables	Spearman Correlation Analysis	
	r	*P* value
Gender	**0.330**	**<0.001**
Age	-0.093	0.129
Diabetes duration	-0.027	0.660
Hypertension	-0.002	0.969
SBP	0.012	0.845
DBP	0.035	0.567
ALT	0.080	0.191
Urea	0.002	0.968
Cr	-0.083	0.175
eGFR	-0.019	0.754
HDL-C	-0.111	0.069
CRP	0.073	0.229
Fasting glucose	**0.125**	**0.040**
HbA1c (%)	0.061	0.315
TG	**0.614**	**<0.001**
TC	**0.342**	**<0.001**
HDL-C	-0.111	0.069
LDL-C	0.054	0.380
N-HDL-C	**0.379**	<**0.001**
TyG index	**0.485**	<**0.001**
NLR	-0.051	0.409
MLR	-0.041	0.500
PHR	**0.123**	**0.044**

SBP, systolic blood pressure; DBP, diastolic blood pressure; ALT, alanine aminotransferase; Cr, creatinine; eGFR, estimated glomerular filtration rate; HDL-C, high-density lipoprotein cholesterol; CRP, C-reactive protein; RC, remnant cholesterol; TG, triglyceride; TC, total cholesterol; HDL-C, high-density lipoprotein cholesterol; LDL-C, low-density lipoprotein cholesterol; N-HDL-C, non-HDL-C; TyG index, triglyceride glucose index; NLR, neutrophil to lymphocyte ratio; MLR, monocyte to lymphocyte ratio; PHR, platelet/HDL-C ratio. P < 0.05 (two-sided) was defined as statistically significant. Bold values indicate statistically significance.

## Discussion

In this study, the relationship was first explored between RC and T2DM - PAD patients. The main conclusions were as follows: (1) RC levels had a positive association with the occurrence and severity of PAD, and RC was independently related to an increased risk of PAD in T2DM patients; (2) diabetic patients with RC levels > 0.64 mmol/L had an elevated risk of developing PAD.

Patients with T2DM and PAD have a cardiovascular mortality risk five times higher than patients with only one disease (17, 18). Hence, effective early screening and identification of T2DM - PAD individuals is crucial (19). Several potential biomarkers have been detected for PAD in diabetic patients, including HMGB 1, OPG, FGF 23, Omentin-1, Cyr61, and Sortilin (20–24). However, there are several limitations to obtaining these data in daily clinical practice. RC can be easily obtained using standard laboratory indices and may have substantial clinical use.

LDL-C is an established risk factor for atherosclerotic cardiovascular disease (ASCVD) (25). However, a high residual risk of CVD persists even in patients whose LDL-C levels meet therapeutic targets after statin therapy, as established by multiple recent meta-analyses (26, 27). RC may be an important contributor of this residual risk (28). In this study, RC levels were significantly higher in the PAD group than in the WPAD group, and LDL-C levels were not significantly different (**Table 1**). The 2019 European Society of Cardiology guidelines recommend that the goal level of LDL be below 1.8 mmol/L with an LDL-C reduction of ≥ 50% from baseline (29). Unfortunately, LDL-C levels failed to meet the established criteria in both groups of patients. In the **Supplementary Material**, the two groups were divided respectively based on the use of statins or not. In the subgroups, LDL-C levels decreased significantly, whereas there was no statistical difference in RC levels. The results indicated that statins did not have a substantial effect on RC levels in T2DM patients with or without PAD (See **Supplementary Tables 1**, **2**). Previous clinical studies have shown that statins reduce RC levels in patients with CAD (30, 31). A prospective cohort with a larger sample size is necessary to see whether statins reduce RC levels in patients with PAD. Comparing the PAD and WPAD groups of patients with LDL-C at the target level, a significant difference in RC levels was found. Elevated RC levels might explain the residual risk of PAD in DM patients with LDL-C level < 1.8 mmol/L (See **Supplementary Table 3**).

In this study, the severity of PAD was graded based on ultrasound measurements, which showed a positive correlation between RC levels and severity (**Figure 1**). Patients were also classified according to the severity of their clinical symptoms using the Fontaine classification (32); however, there was no link between the RC levels and the Fontaine classification. This finding provided more evidence that RC should be promoted in clinical settings alongside ultrasonography results for patient evaluation (See **Supplementary Figure 1**). After adjusting for other factors in the lipid profile using partial correlation analysis (all *P* < 0.001), a significant connection was found between RC and ultrasound grading. (**Table 3**)

The multifactorial regression, excluding the effects of confounding factors, showed that RC was independently associated with T2DM - PAD. This study also demonstrated that age and duration of diabetes were independent risk factors, consistent with previous studies (19). The roles of lipid metabolism and inflammation in atherosclerosis are well-established. It is generally accepted that NLR and MLR can be evaluated as inflammatory markers (33, 34). The platelet to HDL-C ratio as a novel inflammatory index has also garnered attention (35). The research also showed that HDL-C was a protective factor, and that NLR, MLR, and PHR were independent risk factors for PAD (**Table 4**). The ability of RC to predict T2DM - PAD was examined by using ROC curve, and the AUC was 0.727. The cut-off value was 0.64 mmol/L, indicating that diabetic patients with RC > 0.64 mmol/L had an elevated risk of developing PAD.

The TyG index, a surrogate for insulin resistance, is significantly related to the gold standard hyperinsulinemic-orthoglycemic clamp (36) and can be a reliable assessment of insulin resistance in patients. RC has been explored to be linked to insulin resistance (37). TyG index showed a correlation with RC (r = 0.485, *P* < 0.001) (**Table 5**), so it could be speculated that elevated RC levels in T2DM - PAD patients might be mediated by insulin resistance. In addition, one of the key mechanisms of pathogenesis for T2DM - PAD is the hypo-inflammatory response (38). It is worth noting that RC can also cause an inflammatory response, resulting in vascular endothelial damage (5). As shown in **Table 5**, CRP, NLR, MLR, and PHR levels were elevated in the T2DM - PAD individuals, but only PHR was significantly linked to RC (r = 0.123, *P* = 0.044). The correlation between inflammation and RC needs to be further verified by a large-scale investigation.

The fact that RC leads to atherosclerosis is the most likely cause of the link between raised RC levels and an increased risk of PAD (39). As with LDL particles, RC may enter the endothelium, where they are predominantly trapped because of their relatively large size (40), leading to the development of atherosclerosis as a result of cholesterol levels (39). Elevated RC levels are considered a risk factor for endothelial vasodilator dysfunction and can upregulate endothelial expression of endothelial-derived proatherogenic thrombogenic molecules *via* redox mechanisms (41, 42).It was reported that at high glucose concentrations, endothelial cells showed increased expression of low-density lipoprotein receptor 1 (LOX-1), thereby increasing vascular dysfunction (43). Interestingly, RC stimulated NAD(P)H oxidase-dependent superoxide formation and induction of cytokines in human umbilical vein endothelial cells (HUVECs) *via* activation of LOX-1, thereby exacerbating atherosclerosis (44). Furthermore, LOX-1-mediated uptake of RC plays important roles in atherogenesis by inducing LOX-1 expression and vascular smooth muscle cell migration, especially in the context of postprandial hyperlipidemia, diabetes, and metabolic syndrome (45). It could be hypothesized that in patients with DM and PAD, RC might also impact the etiology of PAD by inducing LOX-1 expression. Further studies are needed to determine the specific mechanism of action.

However, this current study also has some limitations. First, this was a retrospective cross-sectional study conducted in a single center, unable to determine the causal relationship between disease and RC. Second, the data were collected from clinical databases, and direct measurement of RC has not yet become a routine test for clinical lipid testing. Therefore, only get the calculated RC levels could be obtained. Calculated and measured RC are closely related (46, 47). Previous studies have shown that calculated RC underestimates the risk of myocardial infarction compared to directly measured RC (48). Nevertheless, calculated RC can be easily obtained from available lipid measurements at no additional cost, and therefore has a strong clinical utility. Third, although the non-fasting RC is critical in the development of atherosclerosis (49), only fasting RC levels were considered, possibly ignoring the possible results of non-fasting RC levels (6).Further prospective studies are required to analyze whether RC accelerates atherosclerosis progression.

## Data availability statement

The original contributions presented in the study are included in the article/**Supplementary Material**. Further inquiries can be directed to the corresponding author.

## Ethics statement

The studies involving human participants were reviewed and approved by [2022] IEC CRYJ 0019. Written informed consent for participation was not required for this study in accordance with the national legislation and the institutional requirements.

## Author contributions

SJ and YiS conceived the study plan and contributed to the revision of the final manuscript. YiS collected, analyzed the data, and finished the manuscript writing. YZ and XB participated in data collection and literature search. WC, LW, MS, YaS, and LZ contributed to the manuscript writing and data interpretation. All authors contributed to the article and approved the submitted version.
