# Supplementary material for: Remnant cholesterol is independently asssociated with an increased risk of peripheral artery disease in type 2 diabetic patients
Source: Front Endocrinol (Lausanne). 2023 Feb 15;14:1111152. doi: 10.3389/fendo.2023.1111152 (PMC9974817; doi:10.3389/fendo.2023.1111152)
Supplement: Supplementary file 1 [file DataSheet_1.pdf]

Supplementary Material

Remnant Cholesterol Is Independently Associated with an Increased Risk of Peripheral Artery Disease in Type 2 Diabetic Patients

Yi Song[1], Ying Zhao[2], Xiangli Bai[3], Wenzhuo Cheng[1], Li Wang[1], Meng Shu[1], Yan Shu[1], Liyin Zhang[1], Si Jin[1]*

[1] Department of Endocrinology, Institute of Geriatric Medicine, Liyuan Hospital, Tongji Medical College, Huazhong University of Science and Technology, Wuhan, China.
[2] Geriatric Medicine Center, Key Laboratory of Endocrine Gland Diseases of Zhejiang Province, Department of Endocrinology, Zhejiang Provincial People's Hospital, Affiliated People's Hospital, Hangzhou Medical College, Hangzhou, China.
[3] Department of Laboratory Medicine, Liyuan Hospital, Tongji Medical College, Huazhong University of Science and Technology, Wuhan, China
***Correspondence:**
Si Jin, M.D, Ph.D., Professor
Department of Endocrinology, Institute of geriatric medicine, Liyuan Hospital, Tongji Medical College, Huazhong University of Science and Technology, 39 Lake Road, East Lake Ecological Scenic, Wuhan, Hubei Province, China.
Tel: 86-027-87543437 Fax: 86-027-87543437 Email: Jinsi@hust.edu.cn

The severity of PAD was assessed using the Fontaine classification, comprising four stages: I - asymptomatic; II - intermittent claudication; III - rest pain; and IV - ischemic ulcers or gangrene (27).

We separated T2DM-PAD and T2DM-WPAD patients based on whether or not they were taking statins to determine whether statin use affected the RC levels. As shown in the Supplementary Table 1 and Table 2, there were no significant differences between the statin and non-statin subgroups in terms of RC. Comparing the PAD and WPAD groups of patients with LDL-C at target levels, a significant difference in RC levels was found. Elevated RC levels might explain the increased risk of PAD in DM patients with LDL-C at target. (See Supplementary Table 3)

Based on the Fontaine classification, we evaluated the degree of PAD disease and then categorized the patients with T2DM - PAD into three subgroups: Fontaine stage II, III, and IV. There was no significant correlation between RC and disease severity in any of the three patient groups.

Supplementary Table 1. Baseline and laboratory findings of T2DM-WPAD patients with statins or without statins

Variables	Non-statins (N=217)	Statins (N=29)	*P* value
Gender (male, %)	121(55.8%)	14(48.3%)	0.447
Age (years)	56±9	57±10	0.153
Diabetes duration (years)	4(1-10)	5(2-10)	0.281
Smoking, n (%)	64(29.5%)	5(17.2%)	0.168

Alcohol, n (%)	54(24.9%)	6(20.7%)	0.621
Hypertension, n (%)	**98(45.2%)**	**22(75.9%)**	**0.002**
Dyslipidemia, n (%)	**73(33.6%)**	**17(58.6%)**	**0.009**
SBP (mmHg)	128(117-140)	122(116-135)	0.153
DBP (mmHg)	**80(72-87)**	**77(68-82)**	**0.016**
ALT, U/L	20.3(14.8-31.2)	20.9(14.3-26)	0.511
AST, U/L	20(16-25.3)	22.1(16.1-27.4)	0.565
Urea, mmol/L	5.54(4.48-6.36)	5.3(4.93-6.43)	0.395
Cr, UMOL/L	63.3(52.4-77.4)	56.8(46.1-74.4)	0.262
eGFR(ml/min/1.73m^2)	102.5(91.4-112.1)	99.7(91-106.7)	0.441
Uric Acid, µmol/L	310.2±87.1	303±86.8	0.853
CRP, mg/L	1.2(0.7-2.4)	1.4(0.8-2.3)	0.939
FPG, mmol/L	**10.56(7.55-14.95)**	**7.97(6.19-13.61)**	**0.015**
HbA1c (%)	8.3(6.8-10)	7.3(6.5-8.9)	0.112
TG, mmol/L	1.54(1.08-2.21)	1.6(1.08-2.2)	0.894
TC, mmol/L	**4.62±1.06**	**3.82±0.93**	**<0.001**
HDL-C, mmol/L	1.12(0.97-1.38)	1.15(0.93-1.37)	0.836
LDL-C, mmol/L	**2.97±0.98**	**2.01±0.72**	**<0.001**
N-HDL-C, mmol/L	**3.52(2.88-4.15)**	**2.54(2.25-3.17)**	**<0.001**
RC, mmol/L	0.55(0.37-0.7)	0.57(0.52-0.87)	0.076
TyG index	7.89(7.27-8.48)	7.55(7.16-8.45)	0.169
Neutrophil ,10^9/L	3.43(2.82-4.46)	3.39(3.01-4.27)	0.900
Lymphocyte, 10^9/L	1.7(1.42-2.05)	1.65(1.38-1.88)	0.432
Monocyte, 10^9/L	0.34(0.28-0.42)	0.37(0.27-0.4)	0.885
Platelet, 10^9/L	207(176-242)	206(189-248)	0.708
NLR	2.05(1.59-2.53)	2.02(1.78-2.72)	0.672
MLR	0.19(0.16-0.25)	0.21(0.17-0.27)	0.391
PHR	176.53(143.06-237.76)	184.21(142.68-235.79)	0.649
Use antidiabetes agents			
Insulin, n (%)	26(12%)	1(3.4%)	0.198
Oral drugs, n (%)	100(46%)	11(37.9%)	
Diet control only, n (%)	62(28.6%)	10(34.5%)	
Insulin + Drugs, n (%)	29(13.4%)	7(24%)	

SBP, systolic blood pressure; DBP, diastolic blood pressure; ALT, alanine aminotransferase; AST, aspartate aminotransferase; Cr, creatinine; eGFR, estimated glomerular filtration rate; CRP, C-reactive protein; FPG, fasting plasma glucose; HbA1c, glycosylated hemoglobin; TG, triglyceride; TC, total cholesterol; HDL-C, high-density lipoprotein cholesterol; LDL-C, low-density lipoprotein cholesterol; N-HDL-C, non-HDL-C; RC, remnant cholesterol; TyG index, triglyceride glucose index; NLR, neutrophil to lymphocyte ratio; MLR, monocyte to lymphocyte ratio; PHR, platelet/HDL-C ratio. P <0.05 (two-sided) was defined as statistically significant. Bold values indicate statistically significance.

Supplementary Table2. Baseline and laboratory findings of T2DM-PAD patients with statins or without statins

Variables	Non-statins (N=215)	Statins (N=55)	*P* value
Gender (male, %)	128(59.5%)	28(50.9%)	0.248
Age (years)	65(59-71)	66(61-69.5)	0.756
Diabetes duration (years)	10(5-18)	10(5-18.5)	0.922
Smoking, n (%)	61(28.4%)	12(21.8%)	0.329
Alcohol, n (%)	57(26.5%)	11(20%)	0.321
Hypertension, n (%)	**126(58.6%)**	**41(74.5%)**	**0.030**
Dyslipidemia, n (%)	**67(31.2%)**	**25(45.5%)**	**0.046**
SBP (mmHg)	133(123-144.5)	130(122-142.5)	0.392
DBP (mmHg)	78(70-85)	76(70-81)	0.143
ALT, U/L	16.6(11.6-22.15)	19.1(14.6-27.25)	0.093
AST, U/L	18(15.65-24.2)	20.7(16.95-25.95)	0.087
Urea, mmol/L	5.93(4.51-7.22)	5.83(4.86-7.15)	0.718
Cr, UMOL/L	70.2(56.3-86)	71.1(59.7-78.3)	0.965
eGFR(ml/min/1.73m^2)	90.9(72.35-104.2)	92.8(71.2-101.3)	0.941
Uric Acid, µmol/L	316.3(243.95-380.2)	324.6(275-372.65)	0.670
CRP, mg/L	2.2(1.1-6.15)	1.9(1-4.5)	0.255
FPG, mmol/L	10.1(8-15.1)	9.59(6.59-13.64)	0.131
HbA1c (%)	8.3(7.2-9.95)	8.1(7.1-9.3)	0.490
TG, mmol/L	1.66(1.14-2.36)	1.66(1.135-2.505)	0.886
TC, mmol/L	**4.68(3.84-5.52)**	**3.82(3.39-4.63)**	**<0.001**
HDL-C, mmol/L	1(0.86-1.16)	1(0.865-1.15)	0.964
LDL-C, mmol/L	**2.72(2.1-3.49)**	**1.98(1.69-2.47)**	**<0.001**
N-HDL-C, mmol/L	**3.58(2.8-4.4)**	**2.73(2.26-3.55)**	**<0.001**
RC, mmol/L	0.76(0.61-1.05)	0.75(0.56-0.99)	0.487
TyG index	7.96(7.38-8.5)	7.85(7.175-8.35)	0.296
Neutrophil ,10^9/L	3.99(3.09-5.13)	3.91(3.32-5.04)	0.895
Lymphocyte, 10^9/L	1.44(1.12-1.81)	1.58(1.26-1.88)	0.154
Monocyte, 10^9/L	0.38(0.3-0.49)	0.4(0.3-0.52)	0.643
Platelet, 10^9/L	212(172-260)	195(169-243)	0.275
NLR	2.66(1.96-3.75)	2.55(1.785-3.56)	0.311
MLR	0.26(0.19-0.36)	0.24(0.19-0.33)	0.497
PHR	204.11(163.25-276.29)	201(152.75-271.76)	0.746
Use antidiabetes agents			
Insulin, n (%)	51(23.7%) a	8(14.5%) a	
Oral drugs, n (%)	98(45.6%) a	18(32.7%) a	

Diet control only, n (%)	18(8.4%) a	5(9.1%) a	
Insulin + Drugs, n (%)	**48(22.3%) a**	**24(43.6%) b**	**0.013**
PAD			
Mild PAD, n (%)	108(50.2%)	29(52.7%)	0.806
Moderate PAD, n (%)	52(24.2%)	11(20.0%)	
Severe PAD, n (%)	55(25.6%)	15(27.3%)	

SBP, systolic blood pressure; DBP, diastolic blood pressure; ALT, alanine aminotransferase; AST, aspartate aminotransferase; Cr, creatinine; eGFR, estimated glomerular filtration rate; CRP, C-reactive protein; FPG, fasting plasma glucose; HbA1c, glycosylated hemoglobin; TG, triglyceride; TC, total cholesterol; HDL-C, high-density lipoprotein cholesterol; LDL-C, low-density lipoprotein cholesterol; N-HDL-C, non-HDL-C; RC, remnant cholesterol; TyG index, triglyceride glucose index; NLR, neutrophil to lymphocyte ratio; MLR, monocyte to lymphocyte ratio; PHR, platelet/HDL-C ratio. *P* <0.05 (two-sided) was defined as statistically significant. Bold values indicate statistically significance. a, b: after applying the chi-square test, different superscripts indicate statistically different categorical variables between the 2 groups.

Supplementary Table3. Demographic and clinical data of WPAD and PAD patients with LDL level < 1.8 mmol/L

Variables	WPAD (N=38)	PAD (N=57)	*P* value
Gender (male, %)	24(63.2%)	37(64.9%)	0.861
Age (years)	**57.5(52-64)**	**65(59-71)**	**<0.001**
Diabetes duration (years)	**6.5(2-10)**	**14(4-20)**	**0.002**
Smoking, n (%)	11(28.9%)	13(22.8%)	0.500
Alcohol, n (%)	13(34.2%)	14(24.6%)	0.307
Hypertension, n (%)	22(57.9%)	37(64.9%)	0.490
Dyslipidemia, n (%)	11(28.9%)	15(26.3%)	0.778
Statins	9(23.7%)	19(33.3%)	0.312
SBP (mmHg)	127(119-142)	132(122-140)	0.494
DBP (mmHg)	79(72-85)	78(72-82)	0.270
ALT, U/L	21.15(14-30.9)	19.1(13.1-29.4)	0.310
AST, U/L	20.1(16.7-25.5)	18.8(16-26.9)	0.679
Urea, mmol/L	5.955(4.86-7.6)	6.04(4.53-7.57)	0.939
Cr, UMOL/L	**67.75(49.9-83.7)**	**76.9(65.5-91.2)**	**0.013**
eGFR(ml/min/1.73m^2)	**98.35(91-106.7)**	**86.1(68.5-99.4)**	**0.005**
Uric Acid, µmol/L	305.7(246.9-377.2)	334.3(287.2-373.9)	0.085
CRP, mg/L	1.4(0.6-2.7)	2(1-6.3)	0.063
FPG, mmol/L	10.615(6.56-15)	8.46(6.36-12.16)	0.277
HbA1c (%)	7.45(6.7-8.9)	7.6(6.7-9.1)	0.779
TG, mmol/L	1.155(0.71-2.19)	1.21(0.82-1.81)	0.979
TC, mmol/L	3.12(2.75-3.52)	3.21(2.95-3.37)	0.403

HDL-C, mmol/L	**1.02(0.8-1.34)**	**0.9(0.73-1.06)**	**0.028**
LDL-C, mmol/L	**1.43(1.26-1.57)**	**1.56(1.36-1.67)**	**0.030**
N-HDL-C, mmol/L	**2.08(1.78-2.31)**	**2.24(2.05-2.44)**	**0.037**
RC, mmol/L	**0.51(0.42-0.7)**	**0.73(0.55-0.9)**	**0.002**
TyG index	7.64(6.97-8.16)	7.34(7.07-8.31)	0.514
Neutrophil ,10^9/L	3.94(2.77-4.67)	4.07(3.02-5.22)	0.511
Lymphocyte, 10^9/L	**1.76(1.37-1.91)**	**1.36(1.05-1.73)**	**0.015**
Monocyte, 10^9/L	0.375(0.29-0.46)	0.41(0.29-0.54)	0.185
Platelet, 10^9/L	203(177-231)	195(160-258)	0.802
NLR	**2.17(1.52-3.12)**	**2.75(1.86-3.78)**	**0.043**
MLR	**0.2(0.16-0.28)**	**0.27(0.21-0.38)**	**0.005**
PHR	186.39(154.55-265.52)	226.85(170-310.71)	0.117
Use antidiabetes agents			
Insulin, n (%)	9(23.7%)	22(38.6%)	0.096
Oral drugs, n (%)	12(31.6%)	13(22.8%)	
Diet control only, n (%)	7(18.4%)	3(5.3%)	
Insulin + Drugs, n (%)	10(26.3%)	19(33.3%)	
PAD			
Mild PAD, n (%)	/	27(47.4%)	/
Moderate PAD, n (%)	/	14(24.6%)	/
Severe PAD, n (%)	/	16(28.1%)	/

SBP, systolic blood pressure; DBP, diastolic blood pressure; ALT, alanine aminotransferase; AST, aspartate aminotransferase; Cr, creatinine; eGFR, estimated glomerular filtration rate; CRP, C-reactive protein; FPG, fasting plasma glucose; HbA1c, glycosylated hemoglobin; TG, triglyceride; TC, total cholesterol; HDL-C, high-density lipoprotein cholesterol; LDL-C, low-density lipoprotein cholesterol; N-HDL-C, non-HDL-C; RC, remnant cholesterol; TyG index, triglyceride glucose index; NLR, neutrophil to lymphocyte ratio; MLR, monocyte to lymphocyte ratio; PHR, platelet/HDL-C ratio. P <0.05 (two-sided) was defined as statistically significant. Bold values indicate statistically significance.

Supplementary Figure1. RC level according to PAD severity based on the Fontaine classification.

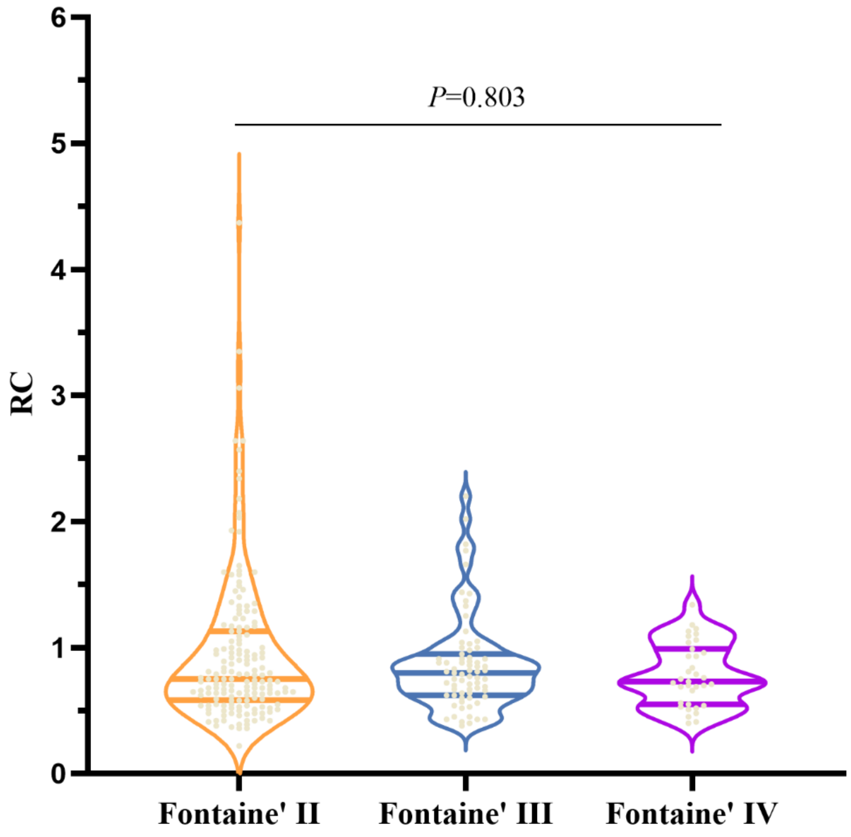

RC, remnant cholesterol. In the violin plot, the three horizontal lines from top to bottom represent the upper quartile, the median, and the lower quartile in order. $P < 0.05$ (two-sided) was defined as statistically significant. Bold values indicate statistically significance.
